# Clinical efficacy and safety of umbralisib, a dual PI3Kδ/CK1-ϵ inhibitor, in treatment of hematologic malignancies

**DOI:** 10.3389/fonc.2025.1591759

**Published:** 2026-01-06

**Authors:** Yunke Zang, Hai-en Cheng, Yanhua Sun, Jingfei Wang, Yuying Zhao, Jingning Yang, Yongping Liu, Yanli Sun

**Affiliations:** 1 School of Medical Laboratory, Shandong Second Medical University., Weifang, China; 2 Department of Hematology, Weifang People’s Hospital, Weifang, China; 3 Neurologic Disorders and Regenerative Repair Laboratory, Shandong Second Medical University, Weifang, China; 4 School of Basic Medical Sciences, Shandong Second Medical University, Weifang, China

**Keywords:** casein kinase-1 ϵ, efficacy, hematologic malignancies, phosphoinositide-3-kinase δ, safety, umbralisib

## Abstract

**Background:**

Umbralisib, a dual PI3Kδ/CK-1ϵ inhibitor, has shown clinical activity in various hematologic malignancies. However, a systematic assessment of its efficacy and safety is still lacking. This study provides a comprehensive evaluation based on current clinical evidence.

**Methods:**

A comprehensive search was conducted in PubMed, Embase, Web of Science, CNKI, and ClinicalTrials.gov for studies involving umbralisib in hematologic malignancies (up to March 14, 2025). Two investigators independently screened eligible studies and extracted data. Efficacy outcomes and adverse events (AEs) were analyzed using meta-analytic methods. The review protocol was registered in PROSPERO (registration number: CRD420251018098).

**Results:**

Umbralisib monotherapy was associated with frequent hematologic AEs such as thrombocytopenia, neutropenia, and anemia, along with common non-hematologic toxicities including diarrhea, nausea, and fatigue. Liver enzyme elevation and diarrhea represented the more severe AEs. Combination therapy showed a distinct AE profile, with infusion reactions and infections being more prominent, but generally demonstrated fewer severe toxicities. In terms of efficacy, monotherapy yielded a modest objective response rate, while combination regimens achieved substantially higher response rates, including improved complete and partial response outcomes.

**Conclusion:**

Umbralisib shows promising efficacy in hematologic malignancies such as MZL, FL, and DLBCL, though its clinical use is limited by frequent AEs. Combination therapy offers better response rates and appears to alleviate some of the severe toxicities seen with monotherapy. Further studies are needed to optimize combination strategies, explore alternative administration routes, and refine dosing approaches.

**Systematic Review Registration:**

https://www.crd.york.ac.uk/PROSPERO/, identifier CRD420251018098.

## 1 Introduction

Hematologic malignancies comprise a heterogeneous group of cancers that continue to impose a substantial global health burden, largely due to their variable clinical behavior and often unsatisfactory long-term outcomes. Among B-cell neoplasms, chronic lymphocytic leukemia (CLL) represents the most common indolent subtype and is characterized by the accumulation of immunologically dysfunctional lymphocytes. Although contemporary registry data indicate a five-year relative survival of approximately 88% overall, prognosis differs markedly across biological and genetic subgroups (1). Follicular lymphoma (FL), another indolent lymphoma, typically follows a relapsing–remitting course; while early-stage disease (stage I) achieves excellent five-year survival rates of up to 97%, outcomes decline considerably in advanced-stage disease, with survival around 83% in stage IV, reflecting the pronounced heterogeneity within FL (2). Marginal zone lymphoma (MZL) generally demonstrates more favorable multi-year progression-free and overall survival, yet patients frequently experience repeated relapses, necessitating ongoing therapeutic intervention (3). In contrast, diffuse large B-cell lymphoma (DLBCL) represents the most common aggressive lymphoma and is associated with substantially poorer outcomes. Population-based analyses report a five-year relative survival of roughly 63%, underscoring the urgency of improving management strategies for this rapidly progressing disease. Together, these variations in disease course, prognosis, and treatment responsiveness highlight the diverse clinical challenges within B-cell malignancies (4).

Over the past two decades, therapeutic innovations have significantly reshaped the treatment landscape. Chemoimmunotherapy, particularly rituximab-containing regimens such as R-CHOP, remains the foundation of care for many patients. However, the introduction of targeted agents, including BTK inhibitors, BCL-2 inhibitors, and PI3K pathway inhibitors, along with monoclonal antibodies, immunomodulatory drugs, CAR-T cell therapies, and bispecific antibodies, has expanded available treatment options and improved outcomes in selected patient groups. These advances have also been accompanied by new patterns of drug resistance and treatment-related toxicities, emphasizing the need for careful therapeutic selection (5).

Phosphoinositide-3-kinase (PI3K), which is implicated in the regulation of cell proliferation and differentiation, has emerged as a promising therapeutic target for hematologic malignancies. The PI3K family consists of four distinct subtypes: α, β, γ, and δ. While the α and β subtypes are ubiquitously expressed in various cell types, the γ and δ subtypes are predominantly found in hematopoietic cells (6). Notably, the δ subtype is a critical component of the B-cell receptor signaling pathway. Aberrant activation of PI3Kδ is frequently observed in B-cell malignancies, making it an attractive target for therapeutic intervention (7, 8).

Umbralisib, a dual inhibitor of PI3Kδ and casein kinase-1ϵ (CK-1ϵ), has demonstrated higher selectivity for PI3Kδ compared to other approved PI3K inhibitors. Specifically, its selectivity for PI3Kδ is more than 1500-fold higher than for the α and β isoforms, and approximately 225 times higher for the γ isoform (9). Moreover, umbralisib uniquely inhibits CK-1ϵ, a key regulator of translational control, which is involved in the translation of the c-Myc oncogene and the regulation of the Wnt5a pathway. CK-1ϵ also plays a role in the immunoregulatory functions of regulatory T cells (Tregs) and is implicated in certain malignancies (10–12).

Preclinical studies in animal models and early-phase clinical trials have shown that continuous treatment with umbralisib has yielded promising objective response rates (ORR) in adults with relapsed or refractory MZL and FL. However, a comprehensive evaluation of umbralisib’s clinical efficacy and safety profile is still needed. This study reviews the clinical trials of umbralisib, focusing on its efficacy and safety profiles.

## 2 Methods

### 2.1 Research design, search strategy, and research selection

This systematic review and meta-analysis was conducted according to PRISMA guidelines. The review protocol was prospectively registered in PROSPERO (CRD420251018098). This study was designed using the participants, intervention, control, outcome, study and design (PICOS) framework. 1) Participants: patients with adult hematologic malignancies. 2) Intervention: treatment with monotherapy or combination therapy with umbralisib. 3) Control: with or without a control group. 4) Outcome: the adverse events (AEs) of all grades and grade ≥ 3, and clinical efficacy indicators, including ORR, complete remission (CR), and partial response (PR), etc. 5) Study design: randomized controlled trial, if not, consider using a single-arm trial. Published research was retrieved from PubMed, Embase, Web of Science, CNKI, and ClinicalTrial.gov using the keyword “umbralisib.” Clinical research on the use of umbralisib to treat hematologic malignancies was collected from the inception to March 14, 2025.

### 2.2 Quality assessment

This study utilizes the methodological index for non-randomized studies (MINORS), which is used for literature quality evaluation for non-random experimental research (13). It has eight evaluation indicators, with each indicator scoring 0, 1, or 2, making the highest possible score 16: 1) whether there is a clearly specified research purpose; 2) whether there is a research object incorporated continuously; 3) whether the data are forward-looking; 4) whether there is a follow-up project fully reflected in the research purpose; 5) whether the evaluation index at the end of the follow-up point is objective; 6) whether the final follow-up time is sufficient; 7) whether the visits are less than 5%; 8) whether the study the design estimates sample quantity. Score 0 indicates that this indicator was not reported, score 1 indicates that this was reported but the data were insufficient, and score 2 indicates that this was reported and had sufficient data.

### 2.3 Inclusion and exclusion criteria

Inclusion Criteria: 1) The research subjects were adult patients diagnosed with hematologic malignancies. The course of the disease, severity, and duration of therapy were not limited. 2) They received either monotherapy or combination therapy with umbralisib. 3) Articles or clinical trials that provided complete clinical efficacy and/or safety data.

Exclusion Criteria: 1) Non-clinical trial articles (such as meta-analyses, reviews, systematic evaluations, conference abstracts, and social editorials). 2) Studies that could not be obtained, extracted, or did not have original data.

### 2.4 Article screening and data extraction

Screening and data extraction were performed independently by two investigators and any disagreements were resolved by a third investigator. EndNote software was used for literature management and data extraction. The extracted content included 1) basic information, including the author, clinical trial registration number, phase, publication year, number of patients, study design and disease type; 2) the data of AEs of all grades and grade ≥ 3, and 3) clinical efficacy indicators, including, but not limited to ORR, CR, PR.

### 2.5 Statistical analysis

In this study, the AEs and survival data were analyzed using Comprehensive Meta-Analysis software, which included the data of AEs of all grades and grade ≥ 3. Confidence intervals (CIs) were set to 95%. Heterogeneity of included articles was analyzed using I^2^ analysis. If I^2^ ≥ 50% and P < 0.05, a random-effects model was chosen in the analysis, otherwise, a fixed-effects model was applied.

## 3 Results

### 3.1 Literature search

**Figure 1:**
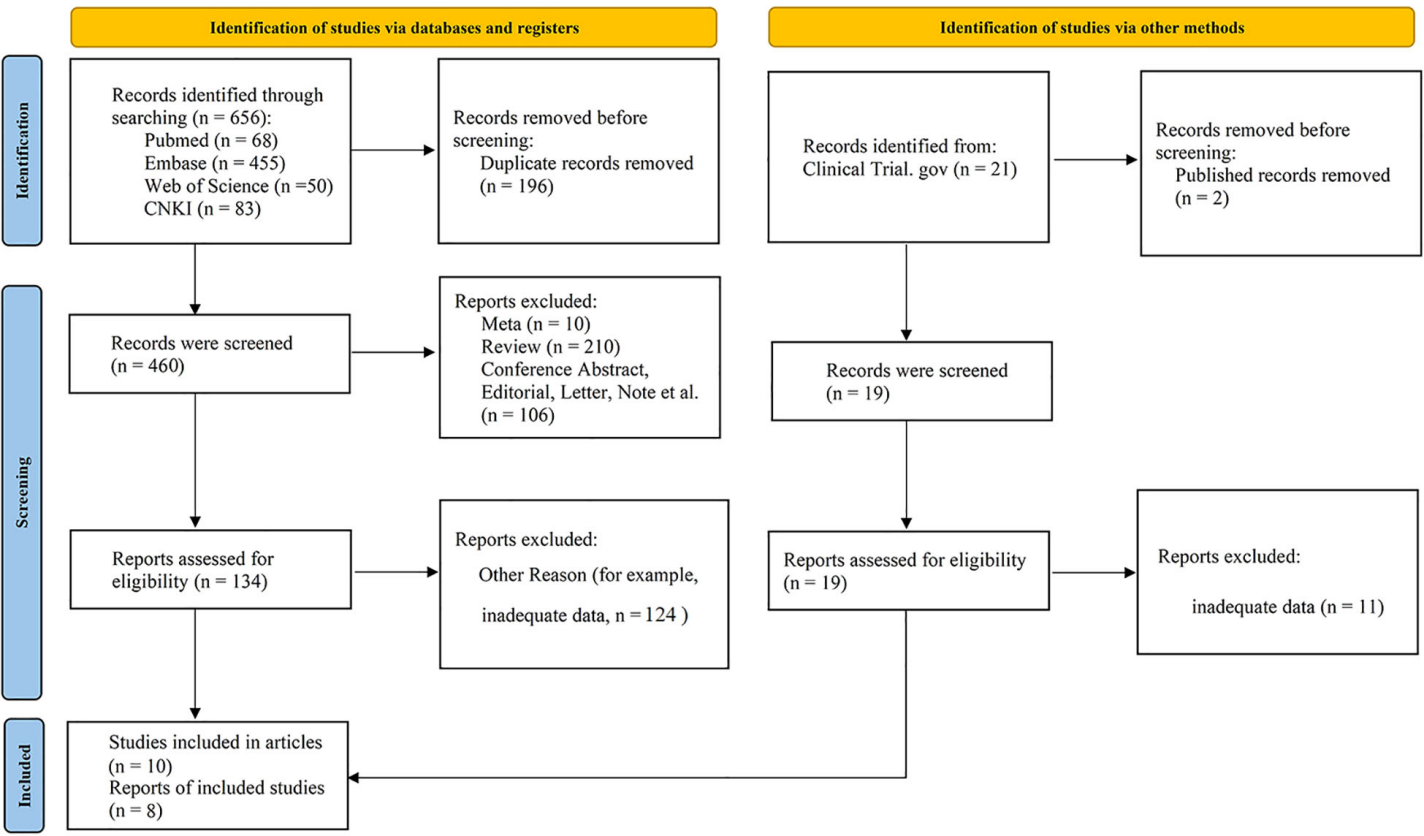
Flow chart of the literature search and screening.

We retrieved 656 related articles and 21 unpublished clinical trials. From preliminary reading of the articles, based on inclusion and exclusion standards, five articles (6, 9, 10, 14, 15) and two clinical trials (NCT03364231 and NCT04163718) on monotherapy, as well as five articles (16–20) and six clinical trials (NCT02656303, NCT04783415, NCT03776864, NCT03801525, NCT04624633 and NCT02612311) on combination therapy, were selected for this study. A total of 1706 patients were included. Details of the screening are shown in **Figure 1** and the basic information of the included articles is listed in **Table 1**.

**Table 1:** Basic information of the selected articles.

Author	Clinical trial registration number	Phase	Publication year	Number of patients	Study design	Disease type
Mato, et al. (2021) (14)	NCT02742090	2	2021	51	Single-arm	CLL
Fowler, et al. (2021) (10)	NCT02793583	2b	2021	208	Single-arm	MZL (n=59), FL (n=117), SLL (n=22)
Davids, et al. (2021) (9)	no mention	1/2	2021	371	Single-arm	FL (n=147), MZL (n=82), DLBCL/MCL (n = 74), CLL/SLL (n = 43), Other (n = 25)
Burris, et al. (2018) (6)	NCT01767766	1	2018	90	Single-arm	CLL (n=24), B-NHL (n=49), HL (n=11), Other (n = 6)
no mention	NCT03364231	2	2022	21	Single-arm	MZL (n=8),WM (n=13)
no mention	NCT04163718	2	2022	12	Single-arm	CLL (n=12)
Zinzani, et al. (2019) (15)	no mention	2	2019	69	Single-arm	Extranodal MZL (n=38), Nodal MZL (n=20), Splenic MZL (n=11)
Nastoupil, et al. (2019) (17)	NCT02006485	1/1b	2019	46	Single-arm	CLL/SLL (n = 23), DLBCL (n = 6), FL (n=8), MCL (n=6), MZL (n=3)
Hill, et al. (2024) (20)	NCT03379051	1/2	2024	46	Single-arm	CLL
Davids, et al. (2019) (18)	NCT02268851	1/1b	2019	42	Single-arm	MCL (n=21), CLL (n=21)
Lunning, et al. (2019) (19)	NCT02006485	1/1b	2019	75	Single-arm	CLL/SLL (n = 22), DLBCL (n = 26), MCL (n=2), Richter (n=1), FL (n=19), MZL (n=5)
Roeker, et al. (2022) (16)	NCT04016805	2	2023	28	Single-arm	CLL
no mention	NCT02656303	2	2023	116	Multi-arm	CLL
no mention	NCT04783415	2	2023	12	Single-arm	MCL
no mention	NCT03776864	2	2023	6	Single-arm	R/R cHL
no mention	NCT03801525	2/3	2024	274	Multi-arm	CLL
no mention	NCT04624633	2	2024	29	Single-arm	CLL
no mention	NCT02612311	3	2024	210	Multi-arm	CLL

CLL, chronic lymphocytic leukemia; MZL, marginal zone lymphoma; FL, follicular lymphoma; DLBCL, diffuse large B-cell lymphoma; MCL, mantle cell lymphoma; SLL, small lymphocytic lymphoma; B-NHL, B-cell non-Hodgkin lymphoma; HL, hodgkin’s lymphoma; WM, Waldenstrom’s Macroglobulinemia.

### 3.2 Quality assessment

The quality of all studies was assessed using the MINORS scale (**Table 1**). All of the studies received high scores but the two clinical trials, NCT03364231 and NCT04163718, which had low scores of 12 (**Supplementary Table S1**). In addition, detailed information on umbralisib dosing schedules for each included study has been provided in **Supplementary Table S2**.

### 3.3 Safety

**Figure 2:**
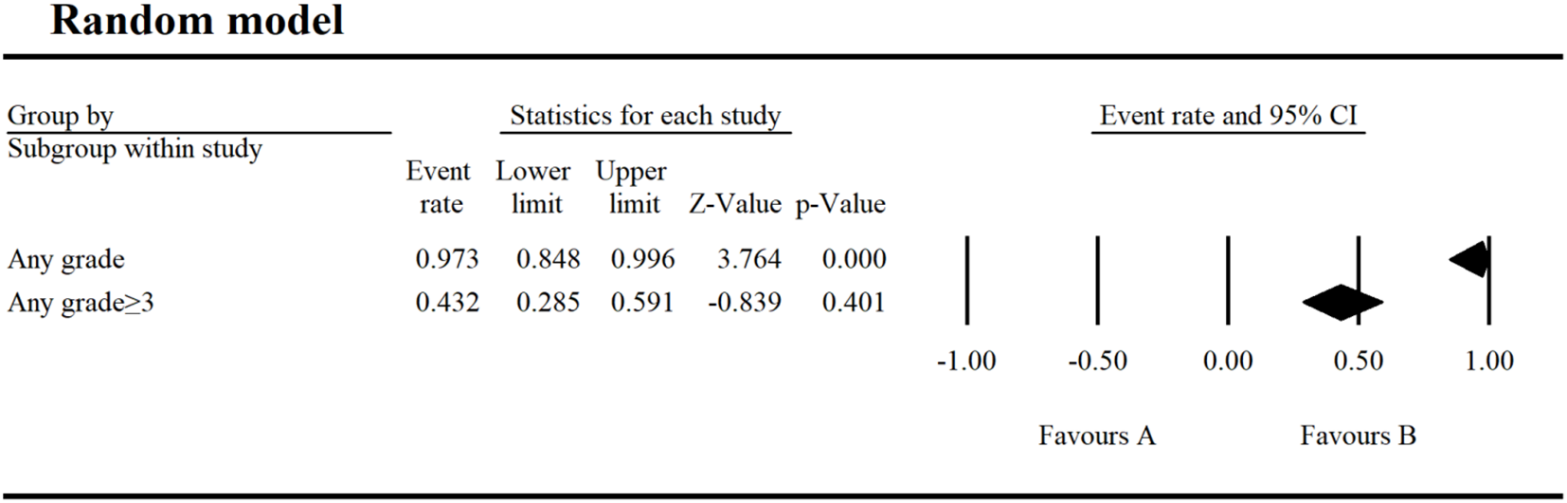
Result of any AEs and any grade ≥ 3 AEs in monotherapy with umbralisib.

**Figure 3:**
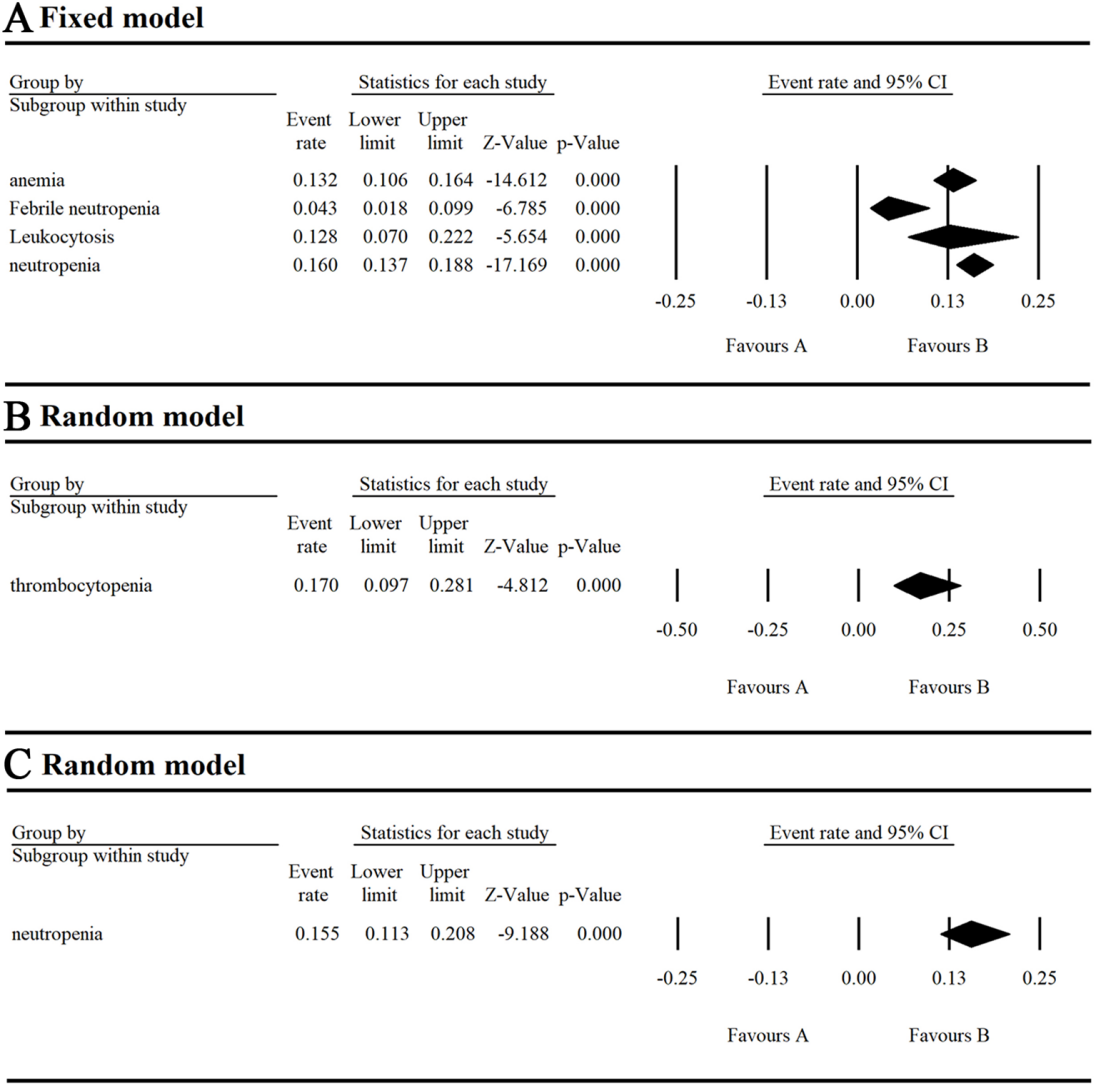
Result of all grade and grade ≥ 3 hematological AEs in monotherapy with umbralisib. **(A, B)** Result of all grade hematological AEs in monotherapy with umbralisib. **(C)** Result of grade ≥ 3 hematological AEs in monotherapy with umbralisib.

**Figure 4:**
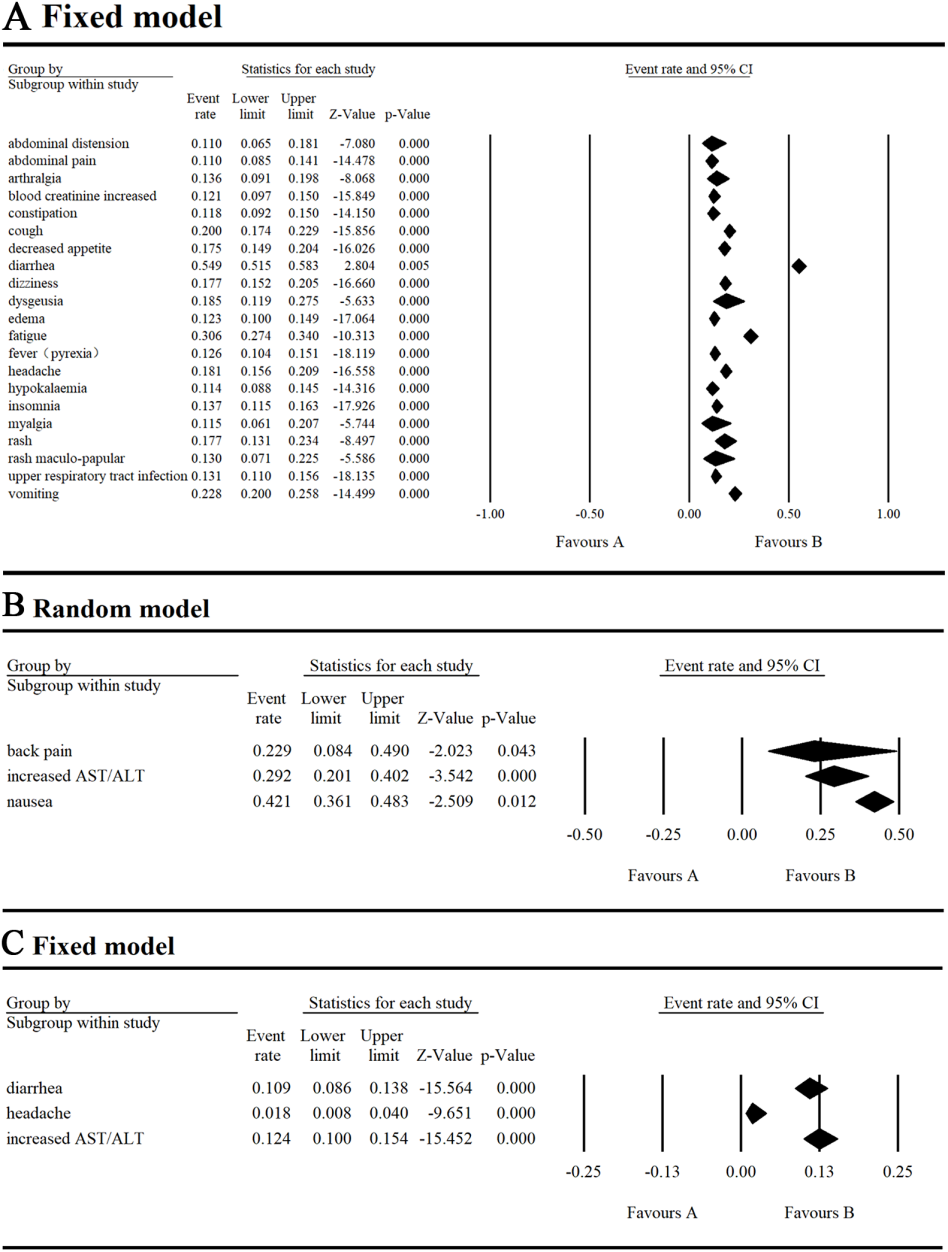
Result of all grade and grade ≥ 3 non-hematological AEs in monotherapy with umbralisib. **(A, B)** Result of all grade non-hematological AEs in monotherapy with umbralisib. **(C)** Result of grade ≥ 3 non-hematological AEs in monotherapy with umbralisib.

**Figure 5:**
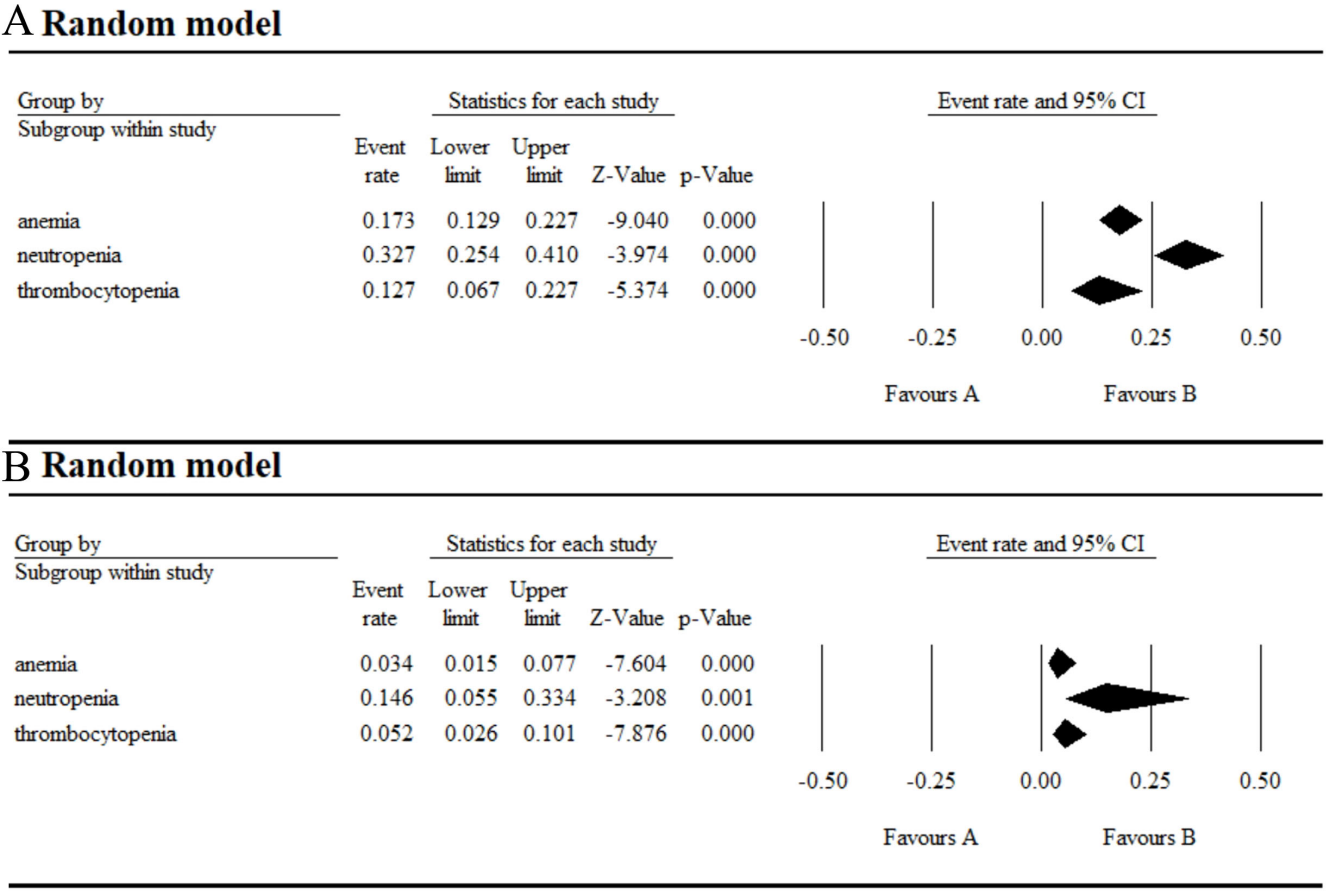
Result of all grade and grade ≥ 3 hematological AEs in combination therapy with umbralisib. **(A)** Result of all grade hematological AEs in combination therapy with umbralisib. **(B)** Result of grade ≥ 3 hematological AEs in combination therapy with umbralisib.

**Figure 6:**
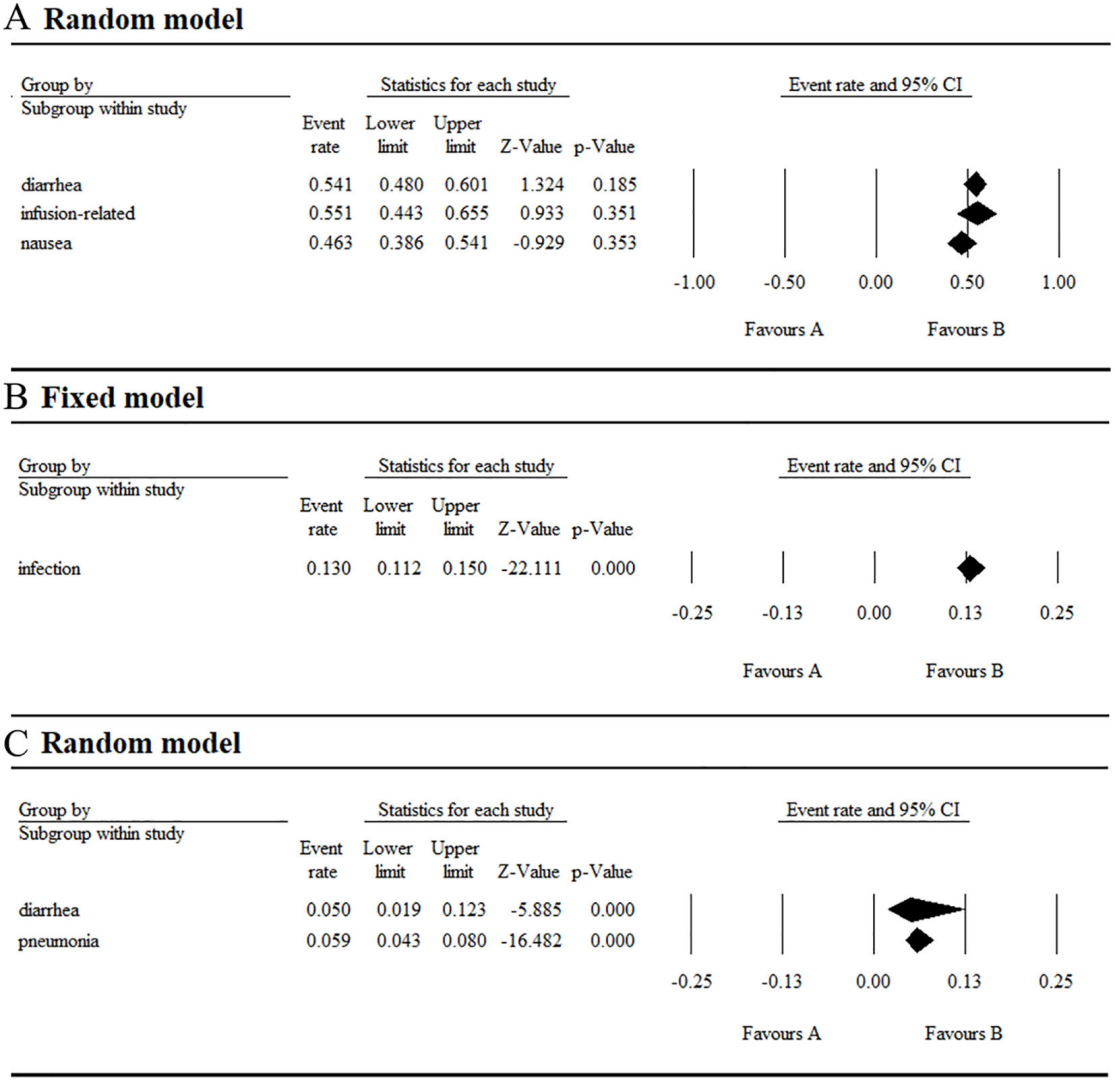
Result of all grade and grade ≥ 3 non-hematological AEs in combination therapy with umbralisib. **(A)** Result of all grade non-hematological AEs in combination therapy with umbralisib. **(B, C)** Result of grade ≥ 3 non-hematological AEs in combination therapy with umbralisib.

The incidence of AEs from 18 selected studies was shown in **Figures 2**–**6**. Umbralisib has been trialed for various hematological diseases, including MZL, FL, DLBCL, SLL, CLL, MCL, B-NHL, Hodgkin’s lymphoma, Waldenstrom’s macroglobulinemia and Relapsed or Refractory Classical Hodgkin (R/R cHL). Data from seven monotherapy studies showed that patients had an AE incidence of 97.3% (95% CI 0.848–0.996) and a grade ≥ 3 AE incidence of 43.2% (95% CI 0.285-0.591). In hematological AEs, the top three all-grade events in umbralisib monotherapy were thrombocytopenia (17%), neutropenia (16%), and anemia (13.2%), with neutropenia being the most common grade ≥ 3 event (15.5%).

For non-hematological AEs, the top three all-grade events were diarrhea (54.9%), nausea (42.1%), and fatigue (30.6%). The top three grade ≥ 3 events were increased AST/ALT (12.4%), diarrhea (10.9%), and headache (1.8%) (**Supplementary Figure S2**). Diarrhea was particularly notable, occurring in over half of the research subjects with an incidence ranging of 0.515-0.583, though the grade ≥ 3 incidence ranged lower. at 0.086-0.138. Neutropenia was the most serious AE, with an incidence of 0.155 (95% CI 0.113-0.208).

In addition, safety evaluations of combination therapy with umbralisib have been performed. In the combination therapy group, the top three all-grade hematological AEs were neutropenia (32.7%), anemia (17.3%) and thrombocytopenia (12.7%), with grade ≥ 3 AEs including neutropenia (14.6%), thrombocytopenia (5.2%), and anemia (3.4%). For all-grade non-hematological AEs, the top three were infusion-related reactions (55.1%), diarrhea (54.1%), and nausea (46.3%), while grade ≥ 3 AEs were mainly infections (13%), pneumonia (5.9%) and diarrhea (5%) (**Supplementary Figure S3**).

Compared to umbralisib monotherapy, combination therapy appears to show preliminary optimization in certain safety indicators while retaining efficacy advantages. Although the potential reduction in specific adverse events such as diarrhea and neutropenia, requires further validation in uniformly designed studies, the overall tolerability profile of combination regimens still provides meaningful insights and supports their potential clinical value within the broader context of PI3Kδ-targeted therapies.

### 3.4 Clinical efficacy

**Figure 7:**
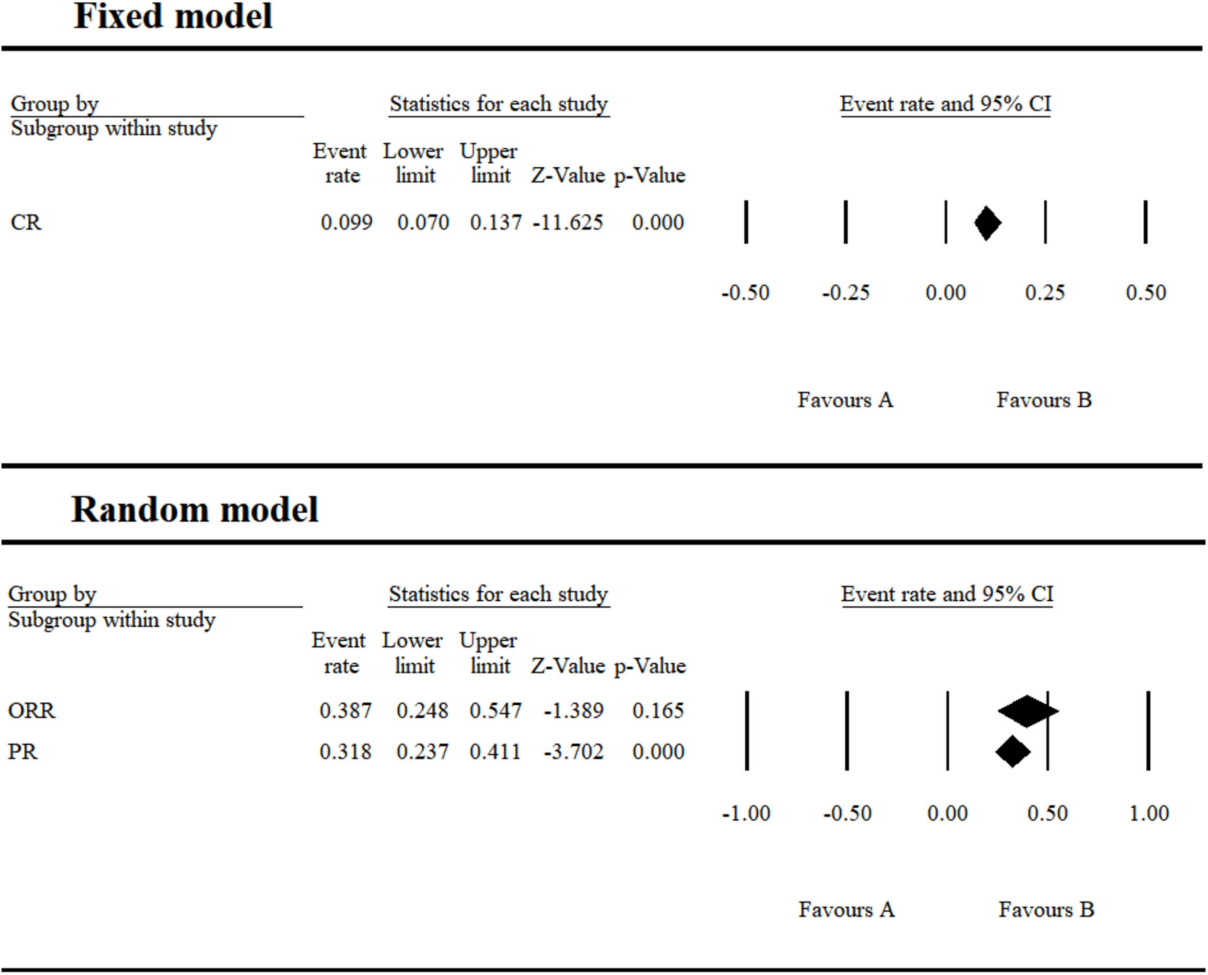
Efficacy of umbralisib monotherapy in the treatment of CLL/SLL.

Current clinical evidence indicates that umbralisib has been evaluated across several hematologic malignancies, including MZL, FL, DLBCL, and SLL/CLL. Because these lymphoma subtypes differ substantially in biological behavior and treatment responsiveness, particularly between indolent entities such as MZL and FL and aggressive diseases such as DLBCL, we did not perform a quantitative pooling of SD or PD outcomes, as combining these heterogeneous endpoints could introduce clinically misleading interpretations. Instead, efficacy findings were summarized descriptively according to individual disease categories. In monotherapy studies, umbralisib demonstrated moderate activity in indolent lymphomas and comparatively limited benefit in aggressive subtypes, with an ORR of 38.7% including a CR rate of 9.9% and a PR rate of 31.8% reported in SLL/CLL (**Figure 7**; **Supplementary Figure S4**).

**Figure 8:**
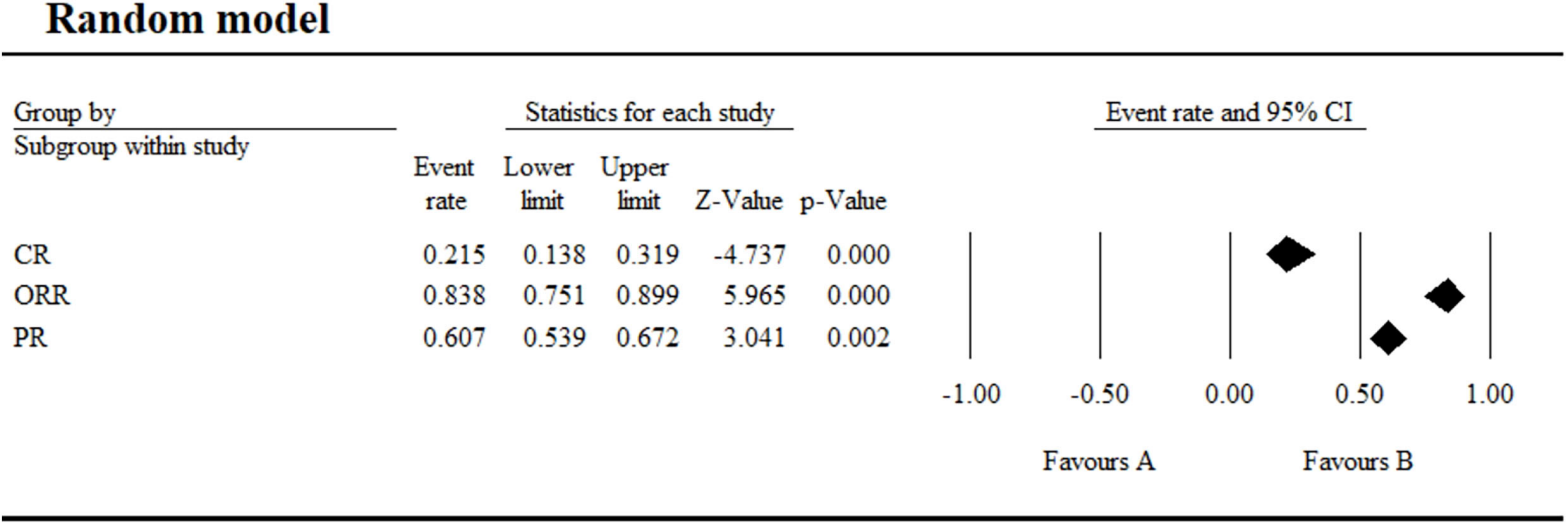
Efficacy of umbralisib combination therapy in the treatment of CLL/SLL.

In contrast, umbralisib-based combination regimens, including U2 (umbralisib plus ublituximab), U2 plus ibrutinib, U2 plus venetoclax, U2 plus acalabrutinib (AU2), and umbralisib plus pembrolizumab, produced substantially enhanced therapeutic activity across CLL/SLL, with a pooled ORR of 83.8% (95% CI 75.1-89.9), a CR rate of 21.5%, and a PR rate of 60.7% (**Figure 8**; **Supplementary Figure S5**). Among these regimens, U2 plus ibrutinib demonstrated the most pronounced efficacy, achieving an ORR of 100% with a CR rate of 36% and a PR rate of 64% in CLL/SLL, representing a considerable improvement relative to umbralisib monotherapy, although differences in disease subtype distribution and patient characteristics should be taken into account when comparing these response estimates.

## 4 Discussion

Umbralisib, a novel targeted therapy for B-cell malignancies, shows higher selectivity for inhibiting P110δ compared to other PI3K subtype inhibitors. Its unique ability to inhibit CK-1ϵ helps to maintain the number and function of regulatory T-cell in patients via the TCF-1 and FoxP3 pathway, reducing immune-mediated side effects in the treatment of hematologic malignancies (21). Additionally, umbralisib preserves IL-10 synthesis while inhibiting Th1/Th2-mediated inflammatory cytokine cascades, lowering immune-mediated complication risks in preclinical CLL studies (22). However, a systematic review of the safety and efficacy of umbralisib remains lacking.

We analyzed AEs in patients with hematologic malignancies undergoing umbralisib monotherapy and combination therapy. Monotherapy resulted in a 97.3% AE incidence and a 43.2% grade ≥ 3 AE incidence, lower than those of copanlisib (84%) (23) and duvelisib (79.44%) (24). The most common hematological AEs were thrombocytopenia (17%), neutropenia (16%), and anemia (13.2%), with neutropenia being the most severe (grade ≥ 3 at 15.5%). Non-hematological AEs were more common, led by diarrhea (54.9%), nausea (42.1%), and fatigue (30.6%), and the most severe was increased AST/ALT (grade ≥ 3 at 12.4%).

In the combination therapy group, the top three all-grade hematological AEs were neutropenia (32.7%), anemia (17.3%), and thrombocytopenia (12.7%), with grade ≥ 3 AEs including neutropenia (14.6%), thrombocytopenia (5.2%), and anemia (3.4%). For all-grade non-hematological AEs, the top three were infusion-related reactions (55.1%), diarrhea (54.1%), and nausea (46.3%), while grade ≥ 3 AEs were mainly infection (13%), pneumonia (5.9%), and diarrhea (5%). Reducing the dose from 800 mg to 400 mg or 200 mg helped to alleviate AE severity (6). Also, preclinical studies indicate that gold nanoparticles as drug carriers can significantly reduce AEs, prolong therapeutic efficacy, and enhance selectivity (25).

Umbralisib monotherapy for SLL/CLL achieved an overall CR of 9.9%, PR of 31.8%, and ORR of 38.7%, suggesting its clinical potential. Combination therapy showed a higher ORR of 83.8% (95% CI 75.1-89.9%), with a CR rate of 21.5% and PR rate of 60.7%. Despite umbralisib’s withdrawal in June 2022 due to a potentially increased risk of mortality and serious AEs, its research continues owing to its remarkable therapeutic effects (26). Ongoing and completed clinical trials (five active, three completed) are investigating umbralisib in combination with other drugs for relapsed/refractory or untreated CLL, MCL, FL, and other hematologic malignancies. Some trials have demonstrated high efficacy and tolerable AEs with umbralisib-based combinations (16, 27–29). In clinical trial NCT03379051, the combination of umbralisib, urituximab, and venetoclax for relapsed/refractory CLL achieved an ORR of 98% and CR of 38%, effectively inhibiting tumor cell signaling pathway activation, rapidly reducing tumor burden, and avoiding tumor lysis syndrome (27, 30). AEs were manageable, with grade 3/4 diarrhea incidence at 14.29% in NCT04624633 and 1.82% in NCT03801525, and with no drug-related deaths reported (16, 27).

For relapsed/refractory mantle cell lymphoma, the combination therapy achieved a CR of 50%, PR of 13%, and ORR of 62% with notably fewer side effects (28). In clinical trial NCT02493530, the combination of umbralisib and ruxolitinib (a JAK1/2 inhibitor) attained an ORR of 56.5% in ruxolitinib-resistant myelofibrosis patients, including two cases of complete remissions. This represents a promising strategy for chronic myelomonocytic leukemia (CMML) patients unresponsive to conventional treatments (29). These findings underscore the urgent need for novel treatment options for patients with hematologic malignancies.

These findings were derived from studies involving heterogeneous lymphoma subtypes, ranging from indolent diseases such as CLL, MZL, and FL to more aggressive entities such as DLBCL. For efficacy, quantitative pooling was feasible only for CLL/SLL due to the availability of sufficient and comparable data, whereas outcomes for other malignancies were summarized descriptively to avoid potentially misleading conclusions. Safety outcomes were analyzed collectively, as adverse events were generally reported consistently across studies. Compared to umbralisib monotherapy, combination regimens appeared to improve certain safety indicators while maintaining efficacy. Overall, combination therapy showed favorable activity in indolent lymphomas and more modest responses in aggressive subtypes, underscoring the need to consider disease biology, patient characteristics, and inter-study variability when interpreting comparative efficacy and safety.

The FDA’s 2022 withdrawal of umbralisib from the U.S. market due to safety concerns further emphasizes these considerations. The trial suggested a possible increased risk of death, prompting the FDA to conclude that the risks outweighed the benefits in patients with MZL and FL. Safety concerns were primarily driven by higher mortality and serious AEs, including infections, diarrhea, hepatotoxicity, and hematologic toxicities (26). Although the studies included in our review generally reported manageable AEs, pooled analyses may not fully capture long-term or population-specific risks. This regulatory context emphasizes the need to balance short-term tolerability against emerging long-term safety considerations.

Future research should prioritize the development of novel combination regimens, the optimization of dosing schedules, and the refinement of administration routes and dosage forms, together with the establishment of appropriate medication guidelines and monitoring strategies. Although umbralisib’s clinical development has included formulation improvements, such as the transition from conventional to micronized tablets, more advanced delivery approaches, including nanoparticle-based carriers, controlled-release formulations, and alternative administration routes, remain largely unexplored. Preclinical and in silico studies indicate that nanoparticle conjugation, for example, using gold nanoparticle platforms, could enhance pharmacokinetics, improve tissue distribution, and reduce off-target toxicity of PI3Kδ inhibitors. Investigating these strategies in preclinical models may improve the therapeutic index of umbralisib, decrease the incidence of adverse events, and expand its clinical applicability. Overall, such efforts have the potential to provide safer and more effective treatment options for patients with hematologic malignancies.

## 5 Limitations

Some inevitable limitations might impact our systematic analysis results. The involved drug is in early clinical trials, and data on clinical efficacy and safety were insufficient due to a limited number of published articles.

## Data Availability

The original contributions presented in the study are included in the article/**Supplementary Material**. Further inquiries can be directed to the corresponding authors.
