# Supplementary material for: Clinical efficacy and safety of umbralisib, a dual PI3Kδ/CK1-ϵ inhibitor, in treatment of hematologic malignancies
Source: Front Oncol. 2026 Jan 6;15:1591759. doi: 10.3389/fonc.2025.1591759 (PMC12815775; doi:10.3389/fonc.2025.1591759)
Supplement: Supplementary file 1 [file DataSheet1.docx]

Supplementary Information

**
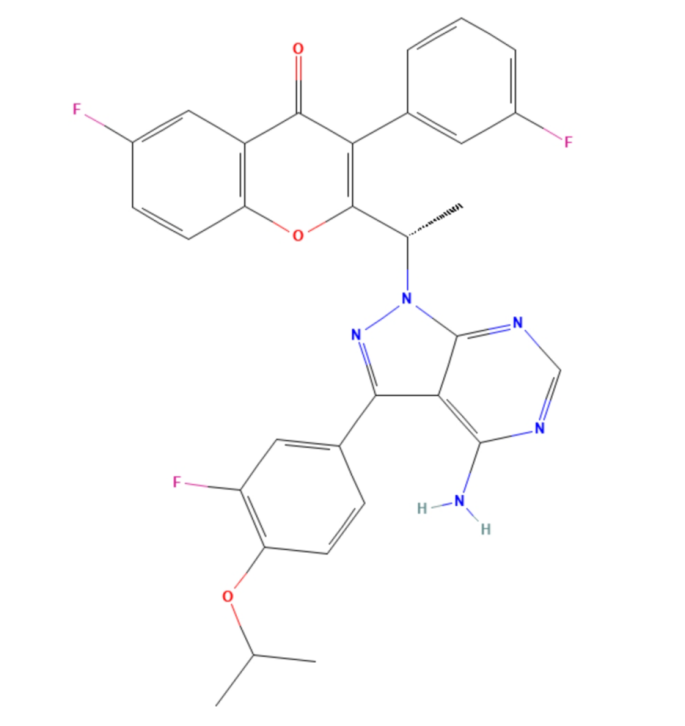
**

**Supplementary Fig. S1** Chemical structure of umbralisib.


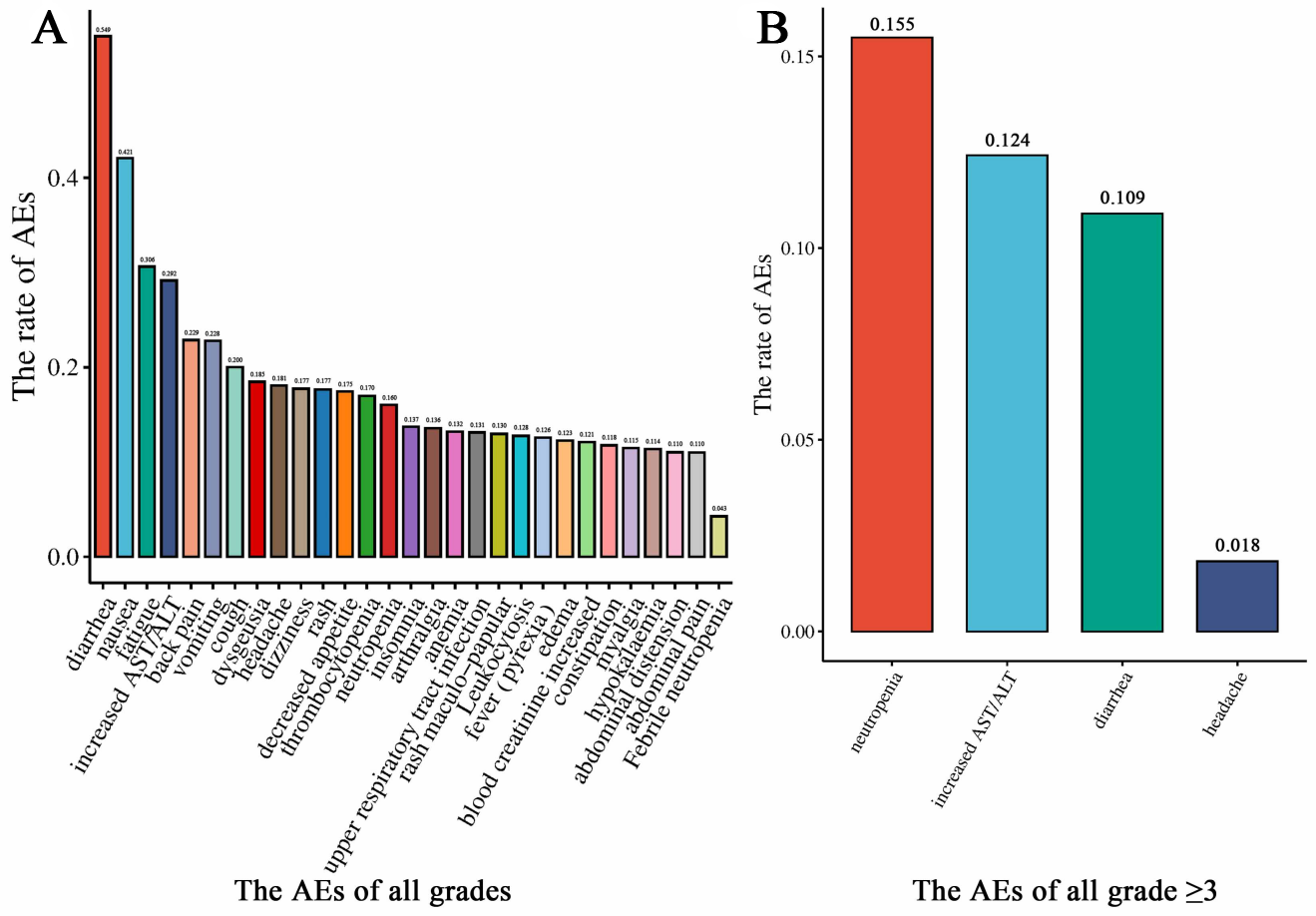


**Supplementary Fig. S2** Result of any AEs and any grade ≥3 AEs in monotherapy with umbralisib.


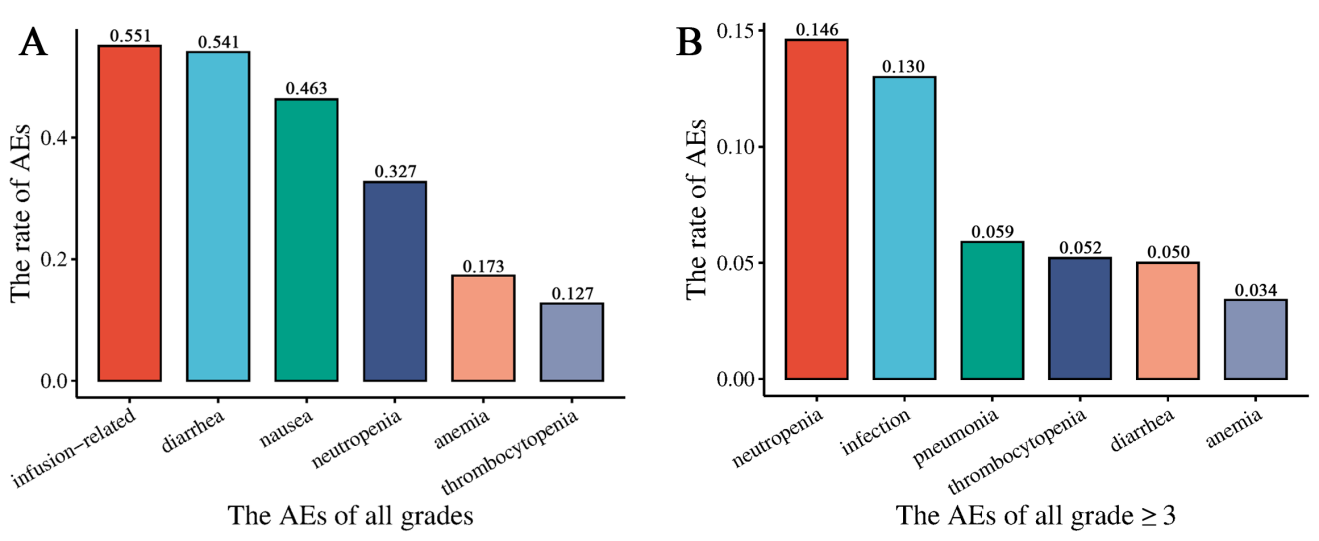


**Supplementary Fig. S3** Result of any AEs and any grade ≥3 AEs in combination therapy with umbralisib.


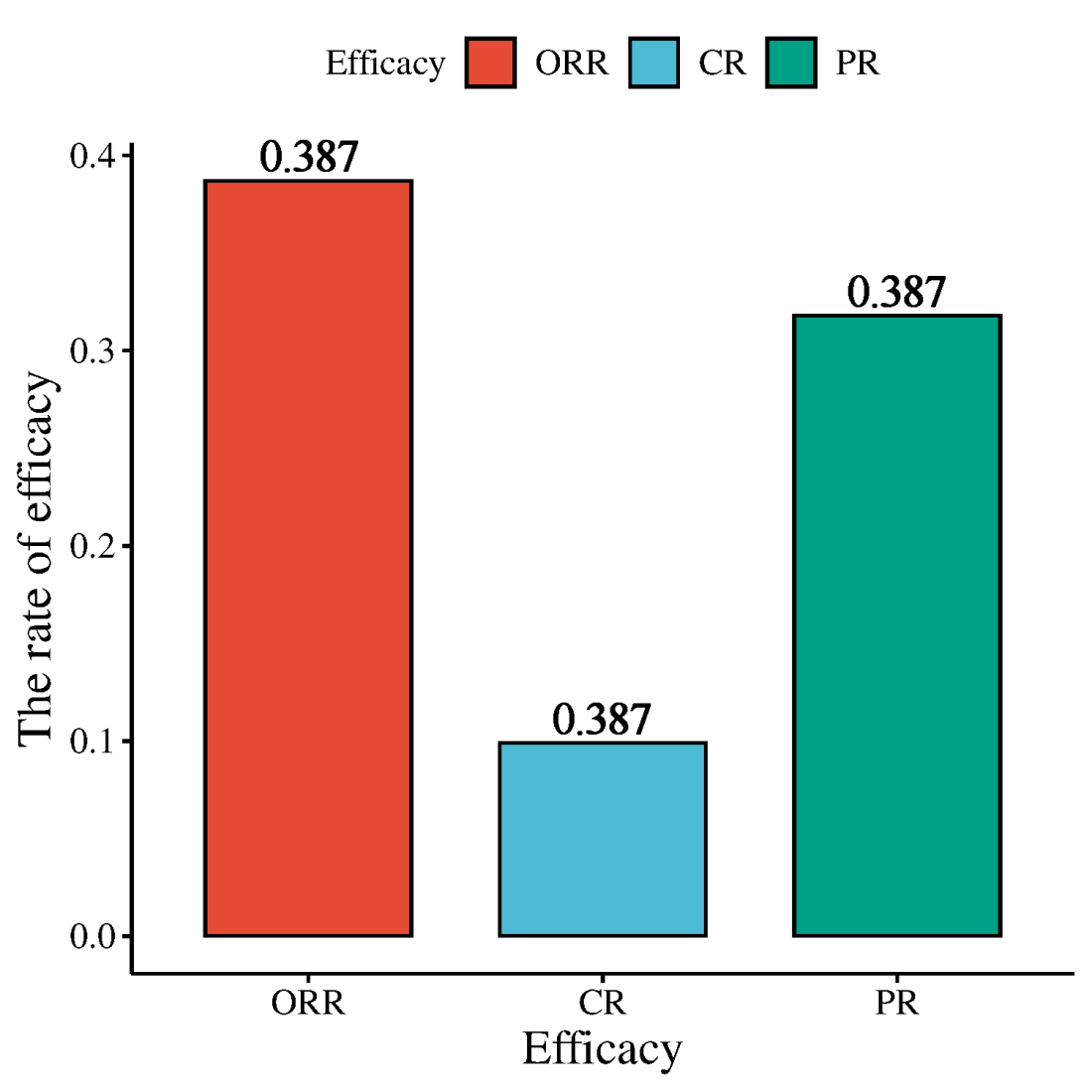


**Supplementary Fig. S4** Efficacy of umbralisib monotherapy in the treatment of hematological malignancies.


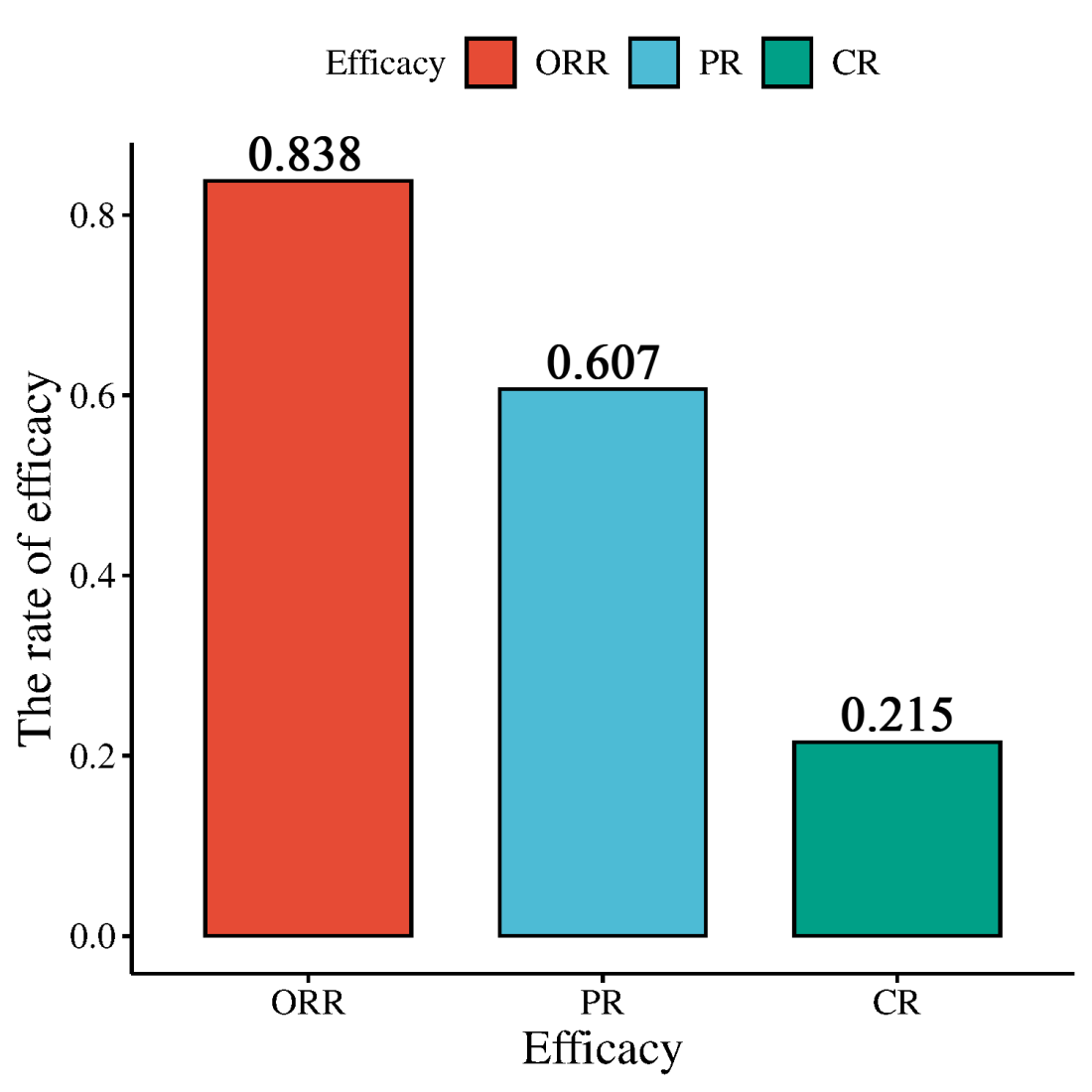


**Supplementary Fig. S5** Efficacy of umbralisib combination therapy in the treatment of hematological malignancies.

# **Supplementary Table S1** The research assessment scores according to MINORS.

| **Author**  **Publication time / NCT_id** | **A clearly stated aim** | **Inclusion of consecutive patients** | **Prospective collection of data** | **Endpoints appropriate to the aim of the study** | **Unbiased assessment of the study endpoint** | **Follow-up period appropriate to the aim of the study** | **Loss to follow up less than 5%** | **Prospective calculation of the study size** | **Total** |
| --- | --- | --- | --- | --- | --- | --- | --- | --- | --- |
| Mato AR (2021) | ★★ | ★★ | ★★ | ★★ | ★★ | ★★ | ★★ | ★★ | 16 |
| Fowler NH (2021) | ★★ | ★★ | ★★ | ★★ | ★★ | ★★ | ★★ | ★★ | 16 |
| Davids MS (2021) | ★★ | ★★ | ★★ | ★★ | ★★ | ★ | ★★ | ★★ | 15 |
| Burris HA 3rd (2018) | ★★ | ★★ | ★★ | ★★ | ★★ | ★★ | ★★ | ★★ | 16 |
| NCT03364231 | ★★ | ★★ | ★★ | ★ | ★ | ★ | ★★ | ★ | 12 |
| NCT04163718 | ★★ | ★★ | ★★ | ★ | ★ | ★ | ★★ | ★ | 12 |
| Zinzani, P (2019) | ★★ | ★★ | ★★ | ★ | ★★ | ★ | ★★ | ★★ | 14 |
| Nastoupil LJ (2019) | ★★ | ★★ | ★★ | ★★ | ★★ | ★★ | ★★ | ★★ | 16 |
| Hill BT (2024) | ★★ | ★★ | ★★ | ★★ | ★★ | ★★ | ★★ | ★★ | 16 |
| Davids MS (2019) | ★★ | ★★ | ★★ | ★★ | ★★ | ★★ | ★★ | ★★ | 16 |
| Lunning M (2019) | ★★ | ★★ | ★★ | ★★ | ★★ | ★★ | ★★ | ★★ | 16 |
| Roeker LE (2022) | ★★ | ★★ | ★★ | ★★ | ★★ | ★★ | ★★ | ★★ | 16 |
| NCT02656303 | ★★ | ★★ | ★★ | ★ | ★ | ★★ | ★★ | ★ | 13 |
| NCT04783415 | ★★ | ★★ | ★★ | ★ | ★ | ★★ | ★★ | ★ | 13 |
| NCT03776864 | ★★ | ★★ | ★★ | ★ | ★ | ★★ | ★★ | ★ | 13 |
| NCT03801525 | ★★ | ★★ | ★★ | ★ | ★ | ★★ | ★★ | ★ | 13 |
| NCT04624633 | ★★ | ★★ | ★★ | ★ | ★ | ★★ | ★★ | ★ | 13 |
| NCT02612311 | ★★ | ★★ | ★★ | ★ | ★ | ★★ | ★★ | ★ | 13 |

# **Supplementary Table S2** Treatment regimen information of the selected articles.

| **Author** | **Clinical trial registration number** | **Treatment regimen** |
| --- | --- | --- |
| Mato, et al. (2021) | NCT02742090 | 800 mg, orally, QD, until progression or toxicity. |
| Fowler, et al. (2021) | NCT02793583 | 800 mg, orally, QD, until disease progression, unacceptable toxicity, or study withdrawal. |
| Davids, et al. (2021) | no mention | 800 mg, orally, QD, until disease progression, unacceptable toxicity, or study withdrawal. |
| Burris, et al. (2018) | NCT01767766 | Orally, QD, the starting dose was 50 mg, followed by dose escalation to 100, 200, 400, 800, 1200, and 1800 mg, until the maximum tolerated dose or the maximal dose level was accrued. |
| no mention | NCT03364231 | 800 mg, orally, QD, until disease progression, unacceptable toxicity or withdrawal from the study whichever occurred first. |
| no mention | NCT04163718 | 800 mg, orally, QD, until removal from study. |
| Zinzani, et al. (2019) | no mention | 800 mg, orally, QD, until disease progression or unacceptable toxicity. |
| Nastoupil, et al. (2019) | NCT02006485 | Treatment consisted of 28-day cycles of ublituximab (900 mg) and ibrutinib (420mg: CLL, 560 mg: B-NHL) combined with escalating doses of umbralisib (400-800 mg) per a 3×3 design, until progression or intolerance. |
| Hill, et al. (2024) | NCT03379051 | Ublituximab: Administered intravenously. The initial dose was 150 mg (Cycle 1, Day 1), followed by 750 mg (Cycle 1, Day 2), and then 900 mg on Cycle 1, Days 8 & 15, and all subsequent cycles (Day 1). The regimen was extended from 3 to 6 cycles after the first 9 patients. Umbralisib: Initiated orally at 600 mg or 800 mg daily from Cycle 1, Day 1. The 800 mg dose was used for Phase 1b, Cohort 2 and all Phase 2 patients. Venetoclax: Started on Cycle 4, Day 1 (or Cycle 2, Day 1 for RT patients) with a standard 5-week dose ramp-up to a target of 400 mg daily. |
| Davids, et al. (2019) | NCT02268851 | Across all cohorts (CLL and MCL), patients received daily oral TGR-1202 and ibrutinib in 28-day cycles until progression or intolerance. TGR-1202: Dose was escalated by cohort (400 mg in Cohort 1, 600 mg in Cohort 2, 800 mg in Cohort 3). Ibrutinib: Dose was 420 mg daily for all cohorts, except for MCL Cohort 1, which received 560 mg daily. |
| Lunning, et al. (2019) | NCT02006485 | The U2 regimen consisted of fixed-dose intravenous ublituximab (900 mg for B-NHL; 600 mg or 900 mg for CLL) administered over a 12-cycle schedule (Cycle 1: Days 1, 8, 15; Cycles 2-6: Day 1; Cycles 7-12: Day 1 every 3rd cycle), alongside continuous daily oral umbralisib, which was initiated at Cycle 1 Day 1 at escalating doses (ranging from 400 mg to 1200 mg using original or micronized formulations) and continued until disease progression, unacceptable toxicity, or study termination. |
| Roeker, et al. (2022) | NCT04016805 | Across all three combination regimens, the U2 backbone (ublituximab 900 mg IV once per cycle for cycles 1–6, then once every three cycles up to 24 cycles, plus umbralisib 800 mg orally once daily through cycles 1–24) was combined with either ibrutinib once daily, venetoclax once daily, or acalabrutinib every 12 hours. |
| no mention | NCT02656303 | Across all arms, the U2 regimen comprised daily oral umbralisib (800 mg) and IV ubiltuximab. Ubiltuximab dosing differed initially: Arms B and D used a initial dose-escalation (150 mg, 750 mg, then 900 mg), while Arm C started at a fixed 900 mg dose. After Cycle 6, all arms received ubiltuximab at 900 mg once every 3 months. |
| no mention | NCT04783415 | Patients receive ublituximab IV over 90 minutes-4 hours on days 1, 8, and 15 of cycle 1 and days 1 of cycles 2-6. Patients also receive acalabrutinib PO BID and umbralisib PO QD on days 1-28. |
| no mention | NCT03776864 | Patients receive pembrolizumab IV on day 1. Treatment repeats every 21 for up to 16 cycles in the absence of disease progression or unacceptable toxicity. Patients also receive umbralisib PO daily on days 1-21 days. Cycles repeat every 21 days in the absence of disease progression or unacceptable toxicity. Pembrolizumab: Given IV, Umbralisib: Given PO |
| no mention | NCT03801525 | Across all three combination regimens (U2 + Ibrutinib, U2 + Venetoclax, U2 + Acalabrutinib), the Ublituximab and Umbralisib (U2) backbone was uniformly dosed: Ublituximab IV at 900 mg once per cycle (Cycles 1-6), then once every three cycles up to Cycle 24, and Umbralisib orally at 800 mg once daily through Cycles 1-24. The third agent varied by regimen: Ibrutinib (once daily), Venetoclax (once daily), or Acalabrutinib (every 12 hours). |
| no mention | NCT04624633 | Based on the provided information, the treatment regimen across both cohorts consisted of acalabrutinib and umbralisib initiated on Cycle 1, Day 1, followed by the addition of ublituximab starting on Cycle 7, Day 1, with all agents continued for a maximum of 24 cycles. Note: Specific dose amounts (mg) for the drugs are not specified in the source text. |
| no mention | NCT02612311 | The study comprised four treatment arms: Arm A (Ublituximab + Umbralisib) and Arm C (Ublituximab monotherapy) followed an identical ublituximab regimen (150 mg, 750 mg, then 900 mg IV in Cycle 1, then 900 mg on Day 1 of Cycles 2-6 and every 3 cycles thereafter), with Arm A adding daily oral umbralisib 800 mg. Arm B (Obinutuzumab + Chlorambucil) received obinutuzumab IV (100 mg, 900 mg, then 1000 mg in Cycle 1, then 1000 mg on Day 1 of Cycles 2-6) plus oral chlorambucil 0.5 mg/kg on Days 1 and 15 of each cycle. Arm D (Umbralisib monotherapy) received daily oral umbralisib 800 mg. All treatments continued until progression, intolerance, or for up to 87 months. |
